# Molybdenum Carbide/Ni Nanoparticles Embedded into Carbon Nanofibers as an Effective Non-Precious Catalyst for Green Hydrogen Production from Methanol Electrooxidation

**DOI:** 10.3390/polym15112430

**Published:** 2023-05-24

**Authors:** Marwa M. Abdel-Aty, Hassan E. Gomaa, Hany Mohamed Abdu, Radwan A. Almasri, Osama M. Irfan, Nasser A. M. Barakat

**Affiliations:** 1 Chemical Engineering Department, Faculty of Engineering, Minia University, Minia 61519, Egypt; 2 Department of Chemistry, College of Science and Humanities, Ad-Dawadmi, Shaqra University, Sahqra 11911, Saudi Arabia; 3 Department of Nuclear Safety Engineering, Nuclear Installations Safety Division, Atomic Energy Authority, Cairo 11765, Egypt; 4 Production Engineering & Design Department, Faculty of Engineering, Minia University, Minya 61516, Egypt; 5 Department of Mechanical Engineering, College of Engineering, Qassim University, Buraydah 51452, Saudi Arabia; 6 Department of Production Engineering, Beni Suef University, Beni Suef 62521, Egypt

**Keywords:** electrospinning, methanol electrooxidation, renewable energy, molybdenum-doped nickel nanofibers, green hydrogen, Taguchi robust design

## Abstract

Molybdenum carbide co-catalyst and carbon nanofiber matrix are suggested to improve the nickel activity toward methanol electrooxidation process. The proposed electrocatalyst has been synthesized by calcination electrospun nanofiber mats composed of molybdenum chloride, nickel acetate, and poly (vinyl alcohol) under vacuum at elevated temperatures. The fabricated catalyst has been characterized using XRD, SEM, and TEM analysis. The electrochemical measurements demonstrated that the fabricated composite acquired specific activity for methanol electrooxidation when molybdenum content and calcination temperature were tuned. In terms of the current density, the highest performance is attributed to the nanofibers obtained from electrospun solution having 5% molybdenum precursor compared to nickel acetate as a current density of 107 mA/cm^2^ was generated. The process operating parameters have been optimized and expressed mathematically using the Taguchi robust design method. Experimental design has been employed in investigating the key operating parameters of methanol electrooxidation reaction to obtain the highest oxidation current density peak. The main effective operating parameters of the methanol oxidation reaction are Mo content in the electrocatalyst, methanol concentration, and reaction temperature. Employing Taguchi’s robust design helped to capture the optimum conditions yielding the maximum current density. The calculations revealed that the optimum parameters are as follows: Mo content, 5 wt.%; methanol concentration, 2.65 M; and reaction temperature, 50 °C. A mathematical model has been statistically derived to describe the experimental data adequately with an *R^2^* value of 0. 979. The optimization process indicated that the maximum current density can be identified statistically at 5% Mo, 2.0 M methanol concentration, and 45 °C operating temperature.

## 1. Introduction

Methanol electrooxidation (MOR) refers to generating hydrogen gas (H_2_) from methanol (CH_3_OH) using an electrochemical cell. This method of producing hydrogen is considered to be “green” because it can be performed by utilizing a renewable energy source, such as solar or wind power, especially since the required cell voltage is small compared to the water electrolysis process, to power the electrochemical reaction, rather than relying on fossil fuels. Additionally, the only byproduct of the reaction is CO_2_, which can be captured and stored to minimize its impact on the environment [1].

Compared to hydrogen production from water electrolysis, MOR has several advantages. Methanol is a more concentrated form of carbon, making it easier to transport and store than water [2]. Water electrolysis is generally considered less efficient than MOR, as the latter requires less energy to generate the same amount of hydrogen [3]. Methanol electrooxidation is generally easier to implement than water electrolysis, as it requires fewer components and has a more straightforward process [4].

Overall, improvement of anode performance in the MOR process can be achieved through different strategies, such as surface modification, bifunctional catalysts, optimized electrode design, proper operating conditions, and the use of membrane electrode assembly [5]. However, the electrode material and design are the main factors that strongly enhance the electrode’s electroactivity. Therefore, it is necessary to consider anode electrocatalysts that increase MOR activity and minimize CO poisoning [5]. Indeed, pure platinum is the most effective electrocatalyst for MOR; however, its high cost and poisoning possibility by CO intermediate limit its wide application [6]. Alloying Pt with another metal, such as Au, Ag, Fe, Pd, Ru, Ir, and Co, was suggested as an effective strategy to improve the electrocatalytic activity toward MOR [7,8,9,10,11]. However, still, the cost dilemma was not resolved.

Transition metals have been widely exploited as cheap and effective catalysts for several chemical reactions. Among the investigated transition metals, nickel-based materials performed satisfactorily in electrocatalytic MORs [12]. However, the corresponding onset potential is still high for use as anodes in the direct methanol fuel cell. However, the low cost and the acceptable performance recommend that nickel-based functional materials are to be exploited in methanol electrooxidation cells to produce green hydrogen gas [13]. Like Pt, pure nickel is not the ideal electrocatalyst composition for MOR; the relatively low electrocatalytic activity is the main issue. Therefore, several metals and non-metals have been utilized as co-catalysts to enhance the electrocatalytic activity of nickel for MOR, such as Co, Pd, N, P, Fe, and others [14,15,16,17].

Molybdenum carbide stands out among the other transition metal carbides because of distinctive qualities and outstanding catalytic abilities in several reactions, such as the selective isomerization of hydrocarbons [18], hydrofining [19], water–gas shift [20], hydrodeoxygenation (HDO) of phenols [21], ammonia synthesis [22], hydrodesulfurization [23], hydrogen evolution reaction [24], and syngas to lower alcohols [25]. As an electrocatalyst, this compound showed a relatively good electrocatalytic performance for MOR and hydrogen electrooxidation reactions [26,27]. As a co-catalyst, molybdenum carbide has been exploited to improve the electrocatalytic activity with an initially active electrocatalyst, such as Pt [27] and Pd [28], toward MOR. However, to our knowledge, molybdenum carbide has not been investigated with nickel to improve the electrocatalytic activity toward MOR.

In electrooxidation reactions, the solid catalyst has to provide a high surface area for the substrate molecules to be adsorbed onto and also facilitates the transfer of electrons between the substrate and the electrode surface, which is crucial for the electrooxidation reaction to occur. The solid catalyst also helps in lowering the reaction’s activation energy, allowing it to proceed more efficiently. Hence, functional electrocatalyst immobilization on nanocarbon supports can tool up dual advantages, utilizing the adsorption capacity of carbon and exploiting the extremely high surface area of the nanostructure. Therefore, several carbon nanostructures have been investigated as supports, including graphene [29], carbon nanotubes [30], and carbon nanofibers [31,32].

The large axial ratio offered by the nanofibrous shape, when compared to other nanostructures, noticeably increases the electron transfer rate and favorably affects the electrocatalytic activity. This is because the nanofibrous structure provides a large surface area for the reaction to take place, increasing the number of active sites available for the electrocatalytic reaction. The large axial ratio also enhances the transport of reactants and products and removes reaction byproducts, leading to a higher rate of electron transfer and improved catalytic activity. Additionally, the nanofibrous morphology enhances the stability of the catalytic materials, reducing the risk of degradation and increasing the longevity of the catalytic process. This is because the fibrous structure provides more stable support for the active species, reducing the risk of corrosion and oxidation of the catalytic materials [31]. These advantages make nanofibrous materials a promising candidate for various electrocatalytic applications, such as energy conversion and storage, environmental remediation, and biosensors [33,34].

As such, the process has many inherent interplaying factors that must be evaluated to capture the optimum conditions that yield maximum conversion within short time intervals (i.e., enhanced process kinetics) at mild conditions with reasonable costs. So, optimizing one factor while the others are fixed at preselected constant levels is unsuitable for properly probing the interactions that may be hidden and requires many runs, implying higher costs and experimentation time [35,36]. Better systematic data mining could be achieved when the variables of interest could be maneuvered over a range of preselected experimental sets. Although the full factorial designs will allow identification of the interactions for a specified set of parameters, it may necessitate conducting many experiments that grow exponentially with the number of concerned parameters [37]. Hence, the researchers have developed a set of research methodologies called fractional factorial designs, intending to reduce the experimental runs to a practical level while keeping the power of probing the possible dynamic interactions [38]. Among those designs, Taguchi constructed a subset of factorial experiments covering many applications and process optimizations with minimal experiments [39]. Accordingly, several nanomaterials have been synthesized based on Taguchi’s robust designs for experimental planning and process variable optimization [40,41,42,43].

This study intended to develop and synthesize a molybdenum carbide/nickel NPs composite incorporated into carbon nanofibers (Mo_2_C/Ni-incorporated CNFs) to exploit the advantages mentioned above of molybdenum carbide and the carbon nanofiber support to maximize MOR yield over this nonprecious electrocatalyst. The fabricated catalyst’s structural, morphological, and textural features have been examined using the respective characterization techniques. The effects of different operating conditions, including chemical composition, methanol concentration, calcination temperature, and oxidation reaction temperature have been investigated and optimized. TM optimization methodology has been utilized to optimize methanol’s electrooxidation using the prepared nanofiber composites.

## 2. Materials and Methods

### 2.1. Materials and Reagents

The employed compounds have been used directly without any further processing. Poly (vinyl alcohol) (PVA), with a molecular weight of 65,000 g/mole, was the polymer used to make the electrospun solution, and it was bought from DC Chemical Co., Seoul South Korea. Molybdenum chloride (MoCl_2_, purity 99.99%) and nickel acetate tetrahydrates (Ni(Ac)_2_, Ni(CH_3_COO)_2_.4H_2_O, 99.99 purity) were purchased from Sigma Aldrich, Seoul, South Korea. The solvent used was deionized water.

### 2.2. Preparation of the Nanofibers and Nanoparticles

A 3:1 weight ratio of 10 weight percent PVA and 20 weight percent Ni(Ac)_2_ aqueous solutions was used to create the Ni(Ac)_2_/PVA aqueous stock. Typically, 10 g of PVA granules were gradually added to 90 mL distilled water under vigorous mixing condition. The mixture was stirred overnight in a hot water bath (~50 °C). After complete polymer dissolution, 1 g of Ni(Ac)_2_ dissolved in 5 mL distilled water was added to the 15 g of the polymer solution. To achieve complete poly(condensation) of the acetate ion, the mixture was agitated for 5 h at 50 °C. Later, several solutions containing 0, 5, 10, 15, 25, and 35 weight percent of molybdenum chloride in comparison to nickel acetate were prepared by dissolving specified quantities of molybdenum chloride in the lowest quantity of deionized water and mixing it with predetermined quantities of the Ni(Ac)_2_/PVA solution. A 20 kV voltage was used throughout the electrospinning technique, and a 15 cm gap was left between the syringe and the rotating drum collector. The electrospun mats were vacuum dried, and then a 5 h holding time calcination procedure was carried out under vacuum at various temperatures (700, 850, and 1000 °C). The sol-gels mentioned above were formulated in the form of very thin film, dried under a vacuum for 24 h at 80 °C, the resulting solids were then thoroughly crushed, and, lastly, they were grounded into nanoparticles and sintered at the aforementioned temperatures. The produced powder was grinded in a ball mill.

### 2.3. Characterization

The characterizations have been performed in the Central lab for Microanalysis and Nanotechnology, Minia University. The nanofibers’ morphology was characterized utilizing SEM and FESEM, Hitachi S-7400, Tokyo, Japan, scanning electron microscope. The chemical structure of the synthesized nanostructures was investigated using Rigaku, Japan, powder X-ray diffractometer (XRD, Rigaku, Tokyo, Japan). Transition electron microscope (TEM) analysis was performed using TEM Phillips CM12 TEM instrument, which was equipped with elemental mapping tool. The sample was first sonicated in pure ethanol for 10 min; then, a copper grid was dipped in the slurry and dried in atmosphere before charging in the instrument. The electrochemical performance of the functional materials was assessed using a potentiostat (VersaStat 4, Ametek Scientific Instrument, Berwyn, PA, USA) in a three-electrode cell consisting of a working electrode, an Ag/AgCl electrode as the reference electrode, and a Pt wire as the counter electrode (CE). The working electrode was prepared by smearing 15 µL of the catalyst ink onto the glassy carbon electrode (GCE) active surface and then dried for 30 min at 80 °C. The ink of the catalyst was formulated by dispersing 2 mg of the active ingredient in a 20 µL Nafion solution and 400 µL isopropanol [44].

### 2.4. Experimental Design

Taguchi’s design was utilized to systemize the experimental investigation of the effects of three independent variables at preselected levels on the response (optimization parameter) by conducting a minimum number of experimental runs. The aim is to improve the relationship between the signal factor and the response with lower sensitivity to the noise factors to obtain a more consistent output. The experimental design selected for methanol electrooxidation is the L16 orthogonal array, with the Mo wt.%, electrooxidation reaction temperature T (°C), and methanol concentration Me (M) being the three input independent variables and the oxidation peak current density mA/cm^2^ as the response. This way, 16 experimental runs were performed, as detailed in Table 1. The results were analyzed employing the Minitab V19.1 statistical software. Since the maximum of the response is desirable, the signal-to-noise ratio S/N, a measure of robustness used to identify control factors that reduce variability in the process by minimizing the effects of uncontrollable factors, was selected as “Larger is better”, Equation (1):

(1) $$\frac{S}{N}=-10Log(\frac{1}{n}\sum \frac{1}{Y^{2}})$$

where *n* is the number of experiments and *Y* is the response.

## 3. Results

### 3.1. Precursors Selection Rationale

**Scheme 1:**
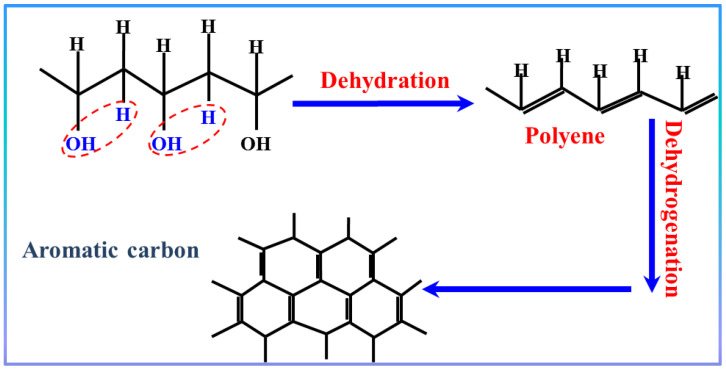
Schematic representation of the PVA’s possible destruction pattern [47].

PVA has significantly more carbon than other vinyl polymers. However, due to the challenges of achieving a suitable form and/or a high carbon yield, utilizing PVA to manufacture carbon nanofibers is uncommon because it melts and decomposes into volatile low molecular weight molecules at low temperatures [45]. Two main approaches have been employed to resolve this issue: (1) pretreatment prior to carbonization and (2) the employment of specific catalysts during heat treatment to enhance graphitization. Both processes include cycling the PVA straight chain, which produces compounds with high melting points that considerably speed up the graphitization process [45]. Scheme 1 depicts the conceptual optimal PVA’s damaging disintegration to obtain the largest yield, showing the possibility of separating aromatic carbon from the PVA straight chain using dehydration and dehydrogenation techniques [46].

The nickel acetate was selected as nickel’s precursor instead of other nickel salts to serve two functions:

1. Creating in situ a reducing environment because it is completely disintegrated under an inert environment to zero-valent nickel (Ni°), as reported by many researchers; upon heating, the acetate anion breaks down to produce reducing gases (CO and H_2_) [48].
2. Having a polycondensation tendency (as illustrated in Equation (2)), which helps preserve the formed nanofibrous structure throughout the calcination process [49].

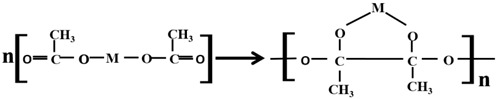
(2)
where M denotes the nickel atom. This way, the electrospinning process was executed efficiently, yielding a good gel that produces good morphology nanofibers.

### 3.2. Characterization of the Prepared Composites

**Figure 1:**
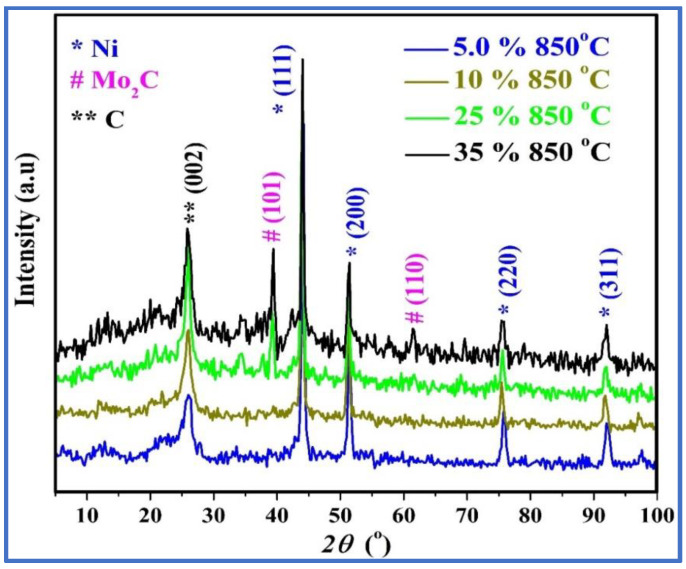
XRD patterns of the calcination products at 850 °C of the electrospun nanofiber mats prepared with different percentages of molybdenum precursor.

X-ray diffraction analysis has been regarded as an effective and reliable method to determine the chemical composition of crystalline materials. XRD patterns were recorded for the obtained products; selected samples’ XRD patterns are shown in Figure 1. The findings corroborated the abovementioned explanation of nickel acetate’s thermal decomposition route. As illustrated, the representative peaks of the Ni° were evident in all formulations, which is confirmed by the strong peaks at 2θ values of 44.30°, 51.55°, 76.05°, and 92.55°, corresponding to the crystal planes with miller indices (111), (200), (220), and (311), respectively (JCDPS# 04-0850). The relatively broad peak at ~26.3°, *d* spacing of 3.37 Å, confirms the presence of graphite-like carbon (graphite-2H, d (002), JCPDS#; 41-1487), demonstrating the successful graphitization of the PVA. Molybdenum was chemically bonded with some of the resultant carbon yielding thermally stable molybdenum carbide (Mo_2_C) with the lowest Mo oxidation number of 2, which may be ascribed to the outgassing of reducing gases during the calcination process [50]. The indexed Mo_2_C peaks (JCPDS; # 35-0787) were confirmed and appeared stronger as the samples’ molybdenum precursor content rose.

**Figure 2:**
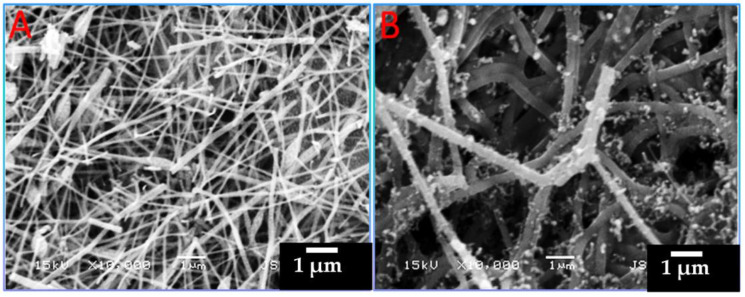
SEM images of the produced Mo_2_C–Ni–C composite nanofibers calcined at 850 °C and obtained from electrospun nanofibers containing 10 (**A**) and 35 (**B**) wt.% MoCl_2_ compared to Ni(AC)_2_.

**Figure 3:**
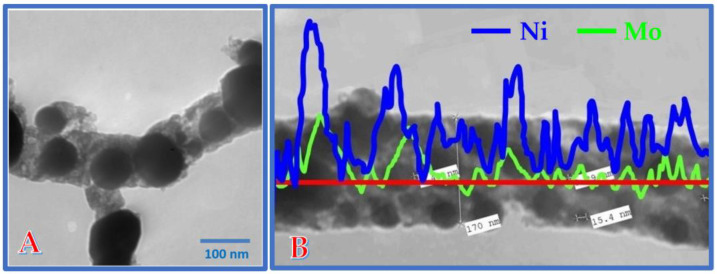
TEM image of Mo_2_C/Ni/graphite composite nanofibers generated from 10% Mo/electrospun solution calcined at 850 °C (**A**) and linear elemental mapping for Ni and Mo along randomly selected (**B**).

SEM images of the samples calcined at 850 °C and obtained from electrospun nanofibers containing 10 (A) and 35 (B) wt.% MoCl_2_ compared to Ni(AC)_2_ are shown in Figure 2A,B. As shown, the images confirm that the nanofibrous morphology of the produced electrospun nanofibers was not destroyed during the calcination at a high temperature. The appeared nanoparticles attaching the main nanofibers at high molybdenum precursor content (Figure 2B) can be attributed to the negative effect of the molybdenum chloride on the poly condensation of the acetate salt, which results in formation of some beads during the electrospinning process. The formed beads are converted to the shown small nanoparticles. Therefore, it is safe to claim that the attached nanoparticles and the main nanofibers have similar composition. It is worth mentioning that all the prepared formulations reveal good nanofibrous morphology (data are not shown). Transmission electron microscopy (TEM) is an approved analytical tool to examine the internal structure of nanostructures. Due to the strong reflection of the applied electron beams, crystalline materials are represented by dark areas in TEM examination. The TEM image of the 10 wt.% nanofibers calcinated at 850 °C (Figure 3A) confirmed that the generated nanofibers are internally structured. Since graphite is observed in the grey matrix during the TEM investigation, it may be assumed that the dark spots represent the equivalent inorganic components of nanofibers. Moreover, an elemental mapping was conducted along with a randomly selected line. As shown in Figure 3B, both nickel and molybdenum were detected in different distributions, which concludes that these two metals do not form an alloy structure. This finding supports XRD results about presence of Mo in the form of molybdenum chloride. Consequently, the physicochemical characterizations concluded that the suggested preparation approach produces Mo_2_C/Ni NPs-incorporated carbon nanofibers as a final product.

### 3.3. Electrochemical Performance

#### 3.3.1. Electroactive Surface Area (ESA)

The nickel-based catalyst surface has to be activated to function as an electrocatalyst for methanol electrooxidation. The activation procedure can be carried out by producing Ni(OOH) species on the surface either simultaneously with the electrooxidation processes or by sweeping in a strongly alkaline solution [51]. The activation process occurs in two primary phases, represented by two regions in the voltammograms. The first region is a negative potential region (at about −650 mV), resulting from nickel hydroxide generation according to Equation (4). It is important to note that the matching peak of this reaction often appears in the first cycle, then disappears [48,52].

Ni + 2OH^−^ ⟷ Ni(OH)_2_ + 2e (3)

The second transition region results from the oxidation of Ni(OH)_2_ to nickel oxyhydroxide (NiOOH), Equation (4), which is completed on the positive region fringe and accompanied by the emergence of a prominent peak [52].

Ni(OH)_2_ + OH^−^ ⟷ NiOOH + H_2_O + e (4)

**Figure 4:**
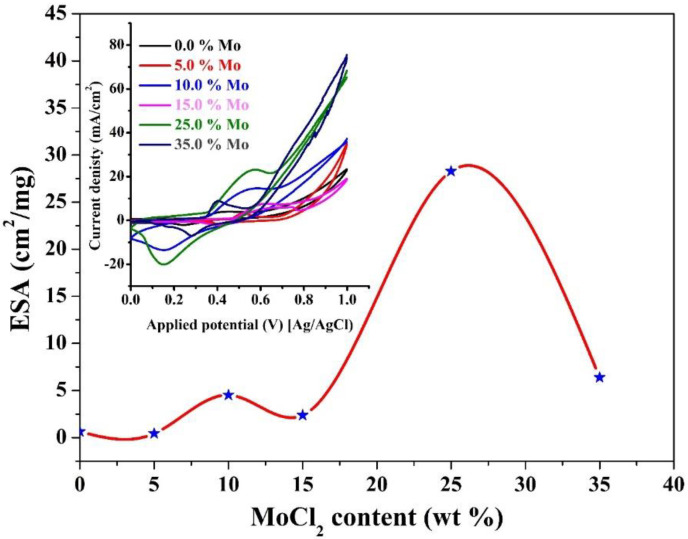
Influence of molybdenum carbide content on the electroactive surface area of the produced Mo_2_C/Ni-incorporated carbon nanofibers. The inset displays the cyclic voltammograms of the prepared nanofibers.

The amount of generated active species and the electroactive surface area closely correlate to nickel-based electrocatalyst activity. The following equation can be used to determine the ESA from the cyclic voltammetry of the activation process [53,54,55].

(5) $$ESA=\frac{Q}{mq}$$

where *Q* (mC) is the charge required for the reduction of NiOOH to Ni(OH)_2_, *m* (mg) is the mass of nickel in the active catalyst, and *q* is the charge associated with the Ni(OH)_2_ monolayer’s formation. The NiOOH transition to Ni(OH)_2_ needs only one electron; hence, *q* can be tuned to 257 C/cm^2^ [32,56,57]. By redrawing the curve as current (mA) versus time (s), the area of the cathodic peak can be used to calculate *Q*. The cyclic voltammograms for nanofibers calcined at 850 °C with different Mo content exhibit distinct peaks at the Ni(OH)_2_/NiOOH transition, the inset in Figure 4.

Figure 4 presents the impact of molybdenum content on the ESA of the prepared nanofibers. As shown, the estimated ESAs for the two formulations were 28.27 and 0.64 cm^2^/mg, respectively. This finding indicates that the ESA of the nanofibers produced from a solution containing 25 weight percent molybdenum precursor enlarged to be 45 times bigger than that of Mo-free nanofibers. However, the results indicated optimizing the Mo content might greatly enhance the ESA. The results also showed that the minor inclusion of the proposed co-catalyst did not noticeably improve the generation of the Ni(OOH) active layer. Pristine and 5 wt.% samples yielded numerically almost identical ESA values, which started exacerbating as the Mo content increased. Nanofibers made from 10–15 wt.% Mo produced ESAs of 4.52 and 2.38 cm^2^/mg, respectively, which are 7 and 3.7 times higher than the Ni/C nanofibers. So, ESA can be improved by amending the initial electrospun solution with higher percentages of MoCl_2_. However, the relationship between the ESA and the Mo content is not linear, with much steep change at the mid-range of Mo percentages (15–25%), giving maxima at ≈25% (≈28 cm^2^/mg) and then diminishing with higher Mo percentages, ≈6.37 cm^2^/mg at 35 wt.% Mo. As such, Mo may affect ESA through specific mechanisms that, more properly represented by a polynomial function, maximize the resultant ESA at an optimum Mo content.

#### 3.3.2. Influence of CNFs Matrix and Mo_2_C Incorporation

**Figure 5:**
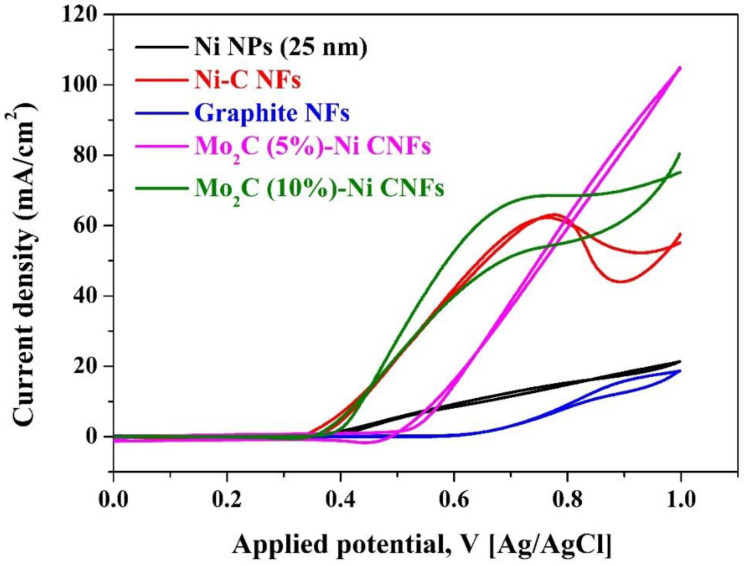
Influences of carbon nanofiber support and Mo_2_C incorporation on the electrocatalytic activity toward methanol oxidation. The experiments were conducted using 3.0 M methanol solution at a scan rate of 50 mV/s and at room temperature.

To properly investigate the impact of the nanofiber matrix and molybdenum carbide incorporation, methanol electrooxidation was carried out in a 3.0 M methanol in 1.0 M KOH solution using different catalyst formulations. Typically, the utilized electrocatalysts were pristine nickel nanoparticles (25 nm average diameter), metal-free carbon nanofibers (graphite nanofibers), Mo_2_C-free Ni NPs-incorporated carbon nanofibers, and two formulations from the proposed Mo_2_C and Ni NPs-incorporated carbon nanofibers, prepared from 5% and 10% samples. The results scientifically support the abovementioned hypothesis about the role of carbon nanofiber matrix and the impact of Mo_2_C in enhancing the electrocatalytic activity toward methanol oxidation. As shown in Figure 5, the lowest performance was related to the metal-free graphite nanofibers, which is an acceptable result due to carbon’s known ignorable electrocatalytic activity toward methanol oxidation. Likewise, the corresponding performance of the pure nickel nanoparticles interestingly showed the importance of the carbonaceous support. As shown in the figure, although nickel is the main catalyst, the absence of adsorbing agent results in unsatisfactory electrode performance.

On the other hand, utilizing a carbon nanofiber matrix with the remaining three formulations enhances the electrodes’ performance. As observed, Ni NPs-incorporated carbon nanofibers reveal good electrocatalytic activity as the maximum current density increases from 15 to 63 mA/cm^2^ for free and carbon-nanofiber-immobilized nickel nanoparticles, respectively. Moreover, exploiting Mo_2_C as a co-catalyst further improved the methanol electrooxidation reaction due to enhancing the electrocatalytic activity of the modified electrodes. However, the performance depends mainly on the co-catalyst content. In detail, for the nanofibers prepared from a 5 wt.% sample, the performance improvement was reflected into enhancing the maximum current density from 63 to 68 mA/cm^2^ and almost no impact on the onset potential. On the other hand, for the nanofibers prepared from 10 wt.% electrospun solutions, there is a sharp increase in the current density, as is shown in the figure; however, there is a little negative impact on the onset potential, which can be neglected for the sake of the high generated current density.

#### 3.3.3. Influence Mo_2_C Content and Methanol Concentration

**Figure 6:**
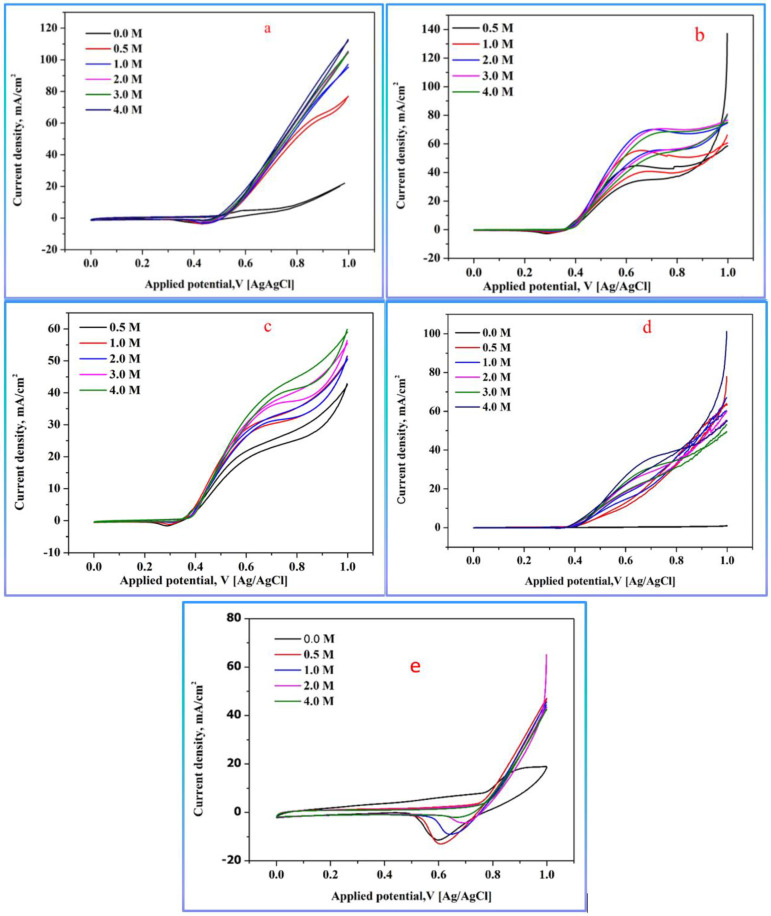
Influence of molybdenum carbide content on the electrocatalytic activity of Mo_2_C–NiCNFs prepared at 850 °C calcination temperature from electrospun solutions having different molybdenum chloride content: 5; (**a**), 10; (**b**), 15; (**c**), 25; (**d**) and 35 wt.%; (**e**), at different methanol concentration and scan rate of 50 mv/s.

The electrocatalytic activity of Mo_2_C/Ni NPs carbon nanofibers has been examined in a methanol solution with a gradual increase in concentration; Figure 6. Cyclic voltammetry was used to assess the catalytic activity of the proposed nanofibers in an alkaline medium in the presence and absence of methanol in a 1.0 M KOH alkaline solution. To investigate the effect of Mo_2_C content, the proposed nanofibers were prepared from electrospun solutions with different amounts of molybdenum chloride salt: 5, 10, 15, 25, and 35 wt.% with respect to the added nickel acetate. Considering the high melting points of nickel (1455 °C) and molybdenum carbide (2687 °C), evaporation of these thermally stable species during the preparation procedure is not expected. Accordingly, the content of the metal carbide co-catalyst can be determined to be 7.12, 13.30, 18.71, 27.73, and 34.94 wt.% with respect to the main catalyst in the nanofibers produced from electrospun solutions having molybdenum chloride salt of 5, 10, 15, 25, and 35 wt.%, respectively. The obtained cyclic voltammograms from the investigated electrodes at different methanol concentrations are shown in Figure 6.

In non-zero-order heterogeneous catalytic reactions, the reaction rate depends on the reactants’ concentration. As the reactants’ concentration increases, the reaction rate also increases. This is because a higher concentration of reactants leads to more collisions between the reactant molecules and the catalyst surface, resulting in more active sites occupied and a higher probability of a successful reaction. However, the reaction rate may plateau or even decrease at high concentrations. This is because the active sites on the catalyst surface become saturated with reactant molecules, and the reaction rate becomes limited by the availability of these sites [58]. This hypothesis is experimentally confirmed, as shown in Figure 6. In other words, with some investigated electrodes, increasing methanol concentration results in increasing the current density up to a certain methanol concentration; after that, increasing methanol concentration did not affect the electrooxidation reaction rate. For instance, for the electrodes prepared from the nanofibers containing 7.12 and 13.3 wt.% Mo_2_C (Figure 6a and 6b, respectively), increasing the methanol concentration by more than 2.0 M did not considerably enhance the generated current density. However, for the nanofibers prepared from the electrospun solution containing 15 wt.% molybdenum precursor (i.e., having 18.25 wt.% co-catalyst; Figure 6c), the generated current density has a direct relation with methanol concentration up to the maximum used methanol concentration limit, 4.0 M. This finding indicates that this sample has numerous active sites compared to the previous ones. Almost the same trend could be observed with the nanofibers having 27.73 wt.% of the utilized metal carbide co-catalyst; Figure 6d. Later on, a greater increase in the co-catalyst content negatively impacts the electrocatalytic activity of the proposed catalyst, as shown in Figure 6e. However, the results indicate also that, although it can be claimed that the number of active sites is high in the nanofibers containing high co-catalyst contents (15 and 25 wt.%) compared to those prepared from low co-catalyst contents (5 and 10 wt.%), the latter has a high activity, which can be attributed to the difference in nature between the active sites in the two formulations.

#### 3.3.4. Effect of Calcination Temperature

Calcination temperature can have a significant effect on the characteristics of the electrocatalysts. Calcination is often used to improve their catalytic activity, stability, and selectivity of electrocatalysts. At higher calcination temperatures, electrocatalysts tend to have higher crystallinity and lower surface area. This is because calcination causes the particles to sinter or fuse together, reducing their overall surface area [59]. However, this can also lead to improved stability and durability of the catalyst. Calcination can also affect the chemical composition of the electrocatalyst. At higher temperatures, certain chemical reactions may occur that can alter the composition of the catalyst [60]. For example, the calcination of a carbon-based electrocatalyst may result in removing oxygen-containing functional groups, which can enhance its electrocatalytic activity [61]. For example, the calcination of platinum-based catalysts at high temperatures can cause the platinum nanoparticles to agglomerate and form larger particles, which can reduce their electrocatalytic activity [62].

**Figure 7:**
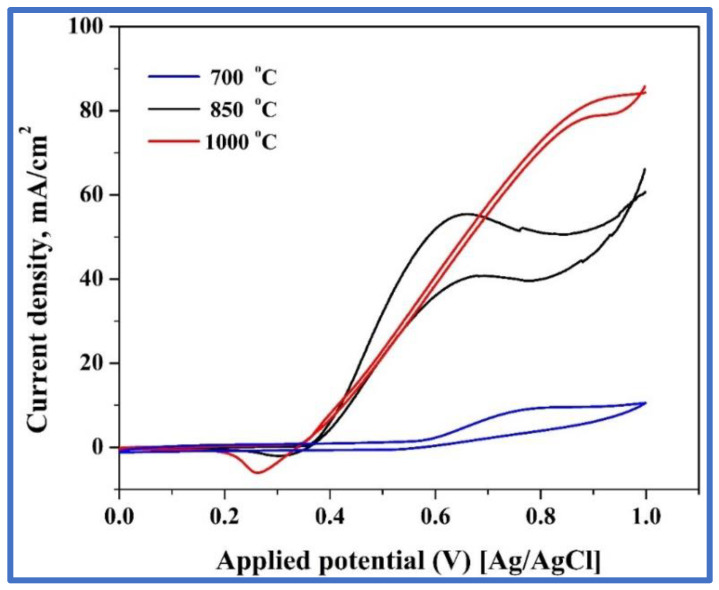
Calcination temperature effect on the electrocatalytic activity of the fabricated NiMo-incorporated carbon nanofibers toward methanol electrooxidation, 5 wt.% Mo_2_C, 1.0 M methanol, scan rate 50 mV/s, and 25 °C.

On the other hand, calcination can also lead to the formation of more active sites on the catalyst surface, improving its performance. Accordingly, the choice of calcination temperature is an important factor in developing electrocatalysts. The optimal calcination temperature depends on the specific characteristics of the catalyst and the desired electrochemical performance. Figure 7 shows voltammograms of 5 wt.% electrodes calcined at different temperatures at 1.0 methanol, exploring the effect of the catalyst’s synthesis temperature on the electroactivity. The electrocatalytic activity of the suggested functional material is enhanced by preparing the proposed nanofibers at a high temperature (850 °C), resulting in large current densities and a clear appearance of the methanol oxidation peak that may be credited to excellent crystallinity at such a high temperature. In contrast, a big difference in the electrocatalytic activity appeared at the treatment temperatures of 700 and 1000 °C.

#### 3.3.5. Effect of Nanostructure Morphology

**Figure 8:**
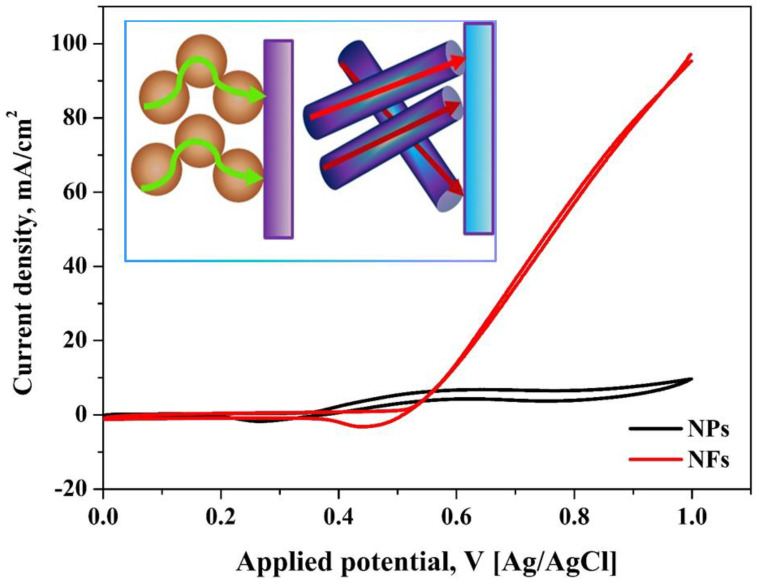
Effect of the nano morphology on the electrocatalytic activity of the proposed MoC_2_/Ni-incorporated carbon nanostructure. The data have been obtained from electrooxidation of 1.0 M methanol in 1.0 M KOH solution at a scan rate of 50 mV/s at 25 °C using nanofibers and nanoparticles prepared from a solution containing 5 wt.% MoCl_2_. The inset displays a conceptual illustration of electron transfer within the nanofibrous and nanoparticulate electrocatalyst.

Electron transfer resistance strongly dependent on the catalyst’s morphology significantly impacts the electrocatalyst’s performance. The axial ratio of a nanostructure refers to the ratio of its length to its width or diameter. This aspect can have a significant impact on the electrocatalytic activity of the material. In general, elongated nanostructures tend to exhibit higher electrocatalytic activity compared to their spherical or short counterparts. The high electrocatalytic activity of elongated nanostructures can be attributed to several factors. Firstly, elongated nanostructures have a larger surface area per unit volume, which can provide more active sites for catalytic reactions. Secondly, the elongated shape can facilitate the diffusion of reactants and products, leading to faster reaction rates. Finally, the elongated shape can promote the formation of specific crystal facets with higher catalytic activity [63]. Moreover, electrocatalyst experiences inherent ohm and interfacial resistances. The latter hinders electrons’ passage through the catalyst interface layer. Accordingly, minimizing the interfacial resistance can strongly enhance the electron transfer process, which shows a positive reflection on the electrocatalytic activity of the catalyst. The long axial ratio characteristic of the nanofibrous morphology produces direct channels to the current collector, considerably reducing the interfacial resistance compared to the nanoparticulate shape. Graphically, the electron paths are illustrated in the inset in Figure 8. As shown in the case of nanoparticles, the electron must pass through several contact sites in a zigzag pass, leading to substantial interfacial resistances. In contrast, the long axial ratio helps create straight routes for electrons to reach the current collector [64]. The idea was demonstrated systematically by producing nanoparticles from the same sol-gel used for electrospinning and calcining them at the same temperature, i.e., 5 wt.% MoCl_2_ at 850 °C. As expected, the two forms exhibited a remarkable difference in the electrocatalytic activities, corroborating the pros of nanofibrous morphology, as shown in Figure 8.

Overall, it can be claimed that utilizing the carbon nanofiber and Mo_2_C as a supporting matric and co-catalyst, respectively, can distinguishably improve the electrocatalytic activity of nickel toward methanol oxidation. To properly support this claim, the observed performance has been compared with some recently reported functional materials; Table 2. As shown in the table, the proposed catalyst outstands itself among the highest activity reported electrocatalysts for methanol oxidation reaction.

#### 3.3.6. Electrooxidation Mechanism

Methanol electrooxidation mechanism has been discussed by several researchers from a long time ago. Based on Fleischmann mechanism, Ni(OOH) is a main reactant in methanol oxidation reactions; the general reactions can be expressed as follows [81,82,83]:

Methanol + H_2_O + NiOOH → Ni(OH)_2_ + Intermediates (6)

Intermediates + NiOOH → Ni(OH)_2_ + Products (7)

Overall: Methanol + H_2_O → CO_2_ + 6H^+^ + 6e (8)

Moreover, a detailed study based on XPS analyses has confirmed the main role of Ni(OOH) in the electrooxidation of methanol over nickel-based electrocatalysts [12]. This active species is regenerated in Equation (4). Therefore, as incorporation of molybdenum carbide with nickel could improve the formed Ni(OOH) layer on the surface of the proposed catalyst (Figure 4), the observed enhanced performance can be understood. The main conflict between the researchers was in interpretation of the intermediates [84]. Based on the density functional theory (DFT), the expected intermediates have been determined to be 16 compounds [85].

### 3.4. Taguchi Optimization of Methanol Electrooxidation Process

The preceding sections evaluated the influences of the different experimental control variables following the one-variable-at-a-time (OVAT) experimental strategy. Reaction temperature, molybdenum content, and methanol concentration remarkably affected the response at different levels, with maxima at certain points. Optimizing those variable levels following Taguchi’s robust design method is deemed suitable. From now on, the design, rationales, and analysis of Taguchi’s experimental design are presented. Molybdenum content, reaction temperature, and methanol concentration are ranked as the most influential factors based on the OFAT results. These control factors were investigated at four levels in 16 experimental runs, as per L16 Taguchi orthogonal array, Table 3. The scheduled experiments were conducted randomly to include the effects of noise factors not considered in the experimental design matrix. The optimization parameter (response) is the electrooxidation peak current density (EOP) expressed with the S/N ratio “larger is better (LIB)”, which corresponds to the desired maximum response.

**Figure 9:**
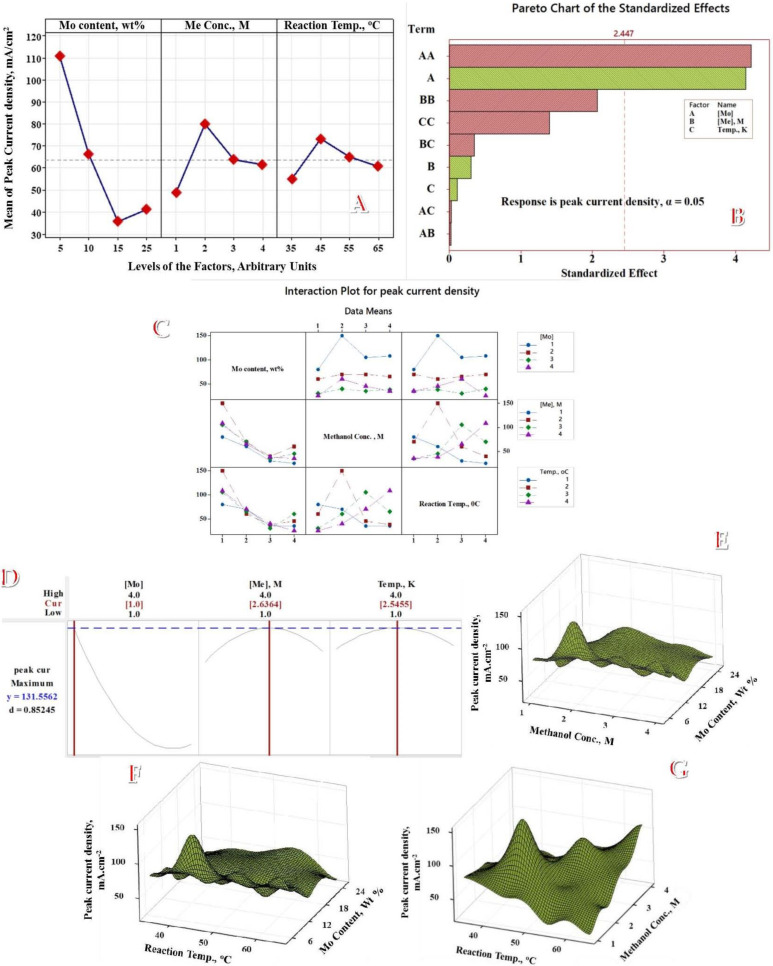
The control factors’ main effects, interaction plots, response optimizer, and 3D surface plots. (**A**) Main effects plot, (**B**) Pareto chart, (**C**) interactions plot, (**D**) response optimizer, (**E**–**G**). 3D surface plots of the three control factors in pairs.

Taguchi’s robust design goes through an optimization process consisting of two consecutive steps. The first step helps identify the control factors that reduce variability using the S/N ratio. The second step identifies the control factors that move the mean to target with minimal or null effects on the S/N ratio. The largest S/N ratios at the respective factor levels and the estimated controlled variables’ rank are given in Table 3. The largest S/N ratio is 40.56 at level one of the factor Mo wt.%, followed by 36.99 and 35.68 for methanol concentration (Me conc.) and temperature, respectively. The main effects plot for EOP is presented in Figure 9, since it is directly readable and interpretable, while the corresponding S/N ratio is given in Table 3.

In the main effects plot (Figure 9a), the molybdenum content is the major playing factor, while the methanol concentration has a milder effect. The Mo wt.% factor exhibited a remarkable decrease from level one to level three, with a steep slope, then re-increased mildly in the reverse, revealing a possible mechanistic change in the material’s structure and its way of action. Inspection of the materials’ XRD pattern in Figure 2 shows that Mo_2_C (101) crystal plane is formed at Mo 25 wt.%, and its peak intensified at the next level, indicating its larger share at higher Mo wt.%. Moreover, the phase Mo_2_C (110) appeared at a Mo content of 35 wt.%. The formation of these phases might be responsible for the steep degradation in the electrocatalytic performance.

In the case of the methanol concentration factor, the trend is different in direction and steepness, since it goes upward from level one to two, showing maxima, then decreases to level three, and, finally, levels off from level three to four, i.e., 2.0 M of methanol gave the largest peak current density. The reaction temperature factor exhibited a very mild effect approaching the quasi-plateau slope. Such a trend demarcates a significant loss in peak current density between levels one and two for Mo wt.% and the reverse for Me conc., indicating the optimum lies around level one of the former and level two of the latter, i.e., 5 wt.% Mo, ≈2 M Me conc., and ≈45 °C.

In conclusion, the process is highly sensitive to the Mo content of the electrocatalyst. The Pareto chart, Figure 9b, compares the proportional influence of each control factor and their mixed effects on the process. The theme reveals the predominance of the Mo content factor on the process and it is worth noting that Mo × Me has negligible weight, marking a reverse effect of both factors upon each other, i.e., both act in a different direction.

ANOVA is a reliable tool for evaluating the descriptive model’s quality as the variation caused by each component is contrasted with the variance caused by random measurement errors. In an ANOVA, a factor’s significance is shown by a *p*-value less than 0.05; if it is greater than 0.05, the optimization parameter has lower sensitivity to the changes in this factor [86]. Table 4 shows the ANOVA table based on S/N data to determine the significance of each parameter in the methanol electrooxidation process.

The ANOVA demonstrates the relative relevance of each parameter using the sequential (Seq) and adjusted (Adj) sum of squares, with the parameter with the highest SS having the main effect. The ANOVA results reinforce the mentioned above findings (Table 4). The molybdenum content variable has an F-value of 75.9, a *p*-value of 0.00, and the highest SS value of 235.36, indicating the statistical significance of this factor changes on the optimization parameter. The methanol concentration ranked second, with an F-value of 11.89, a *p*-value of 0.006, and an SS value of 36.88. The reaction temperature has a small F-value, 1.9, with a *p*-value greater than 0.05, indicating it is statistically insignificant.

Moreover, the interactions between the three control variables depicted in Figure 9c strengthen the ANOVA’s inferences probing which levels contribute most. There were no interactions between Mo wt.% and the other control variables at the first two levels (5 and 10 wt.%), which confirms the dominance of the Mo content control variable over the others leveraging appreciable methanol electrooxidation, i.e., the highest obtained current density. At the next levels (15 and 25 wt.%), the interactions appeared weak and plateaued, which may be explained as, at these levels of the Mo content factor, the dominance of this factor on optimization parameters decayed and the other factors expressed their effects. The other factors’ interactions appeared much stronger, indicating the sensitivity of the optimization parameter to their changes, with the second level (2 M) of methanol concentration expressing significant improvement in the process’s performance. Figure 9d shows the optimum conditions’ positioning using the response optimizer feature. The drawn optimum conditions are Mo content: 5 wt.%, methanol concentration: 2.65 M, and reaction temperature: 50 °C.

The objective is to optimize all interplaying control factors to effect maximum methanol electrooxidation at the lowest possible Mo content, methanol concentration, and reaction temperature, which might enhance the process efficiency and economics and reduce the effluent load. The interaction theme for every two factors is portrayed in the surface plot (Figure 9e–g). The trend emphasizes the dominance of the Mo content at its lower levels upon all other control factors, as indicated by individual maxima (crest) corresponding to the lower fringe of Mo content, Figure 9e,f. The 3D surface plot of reaction temperature and methanol concentration experiences a taller crest corresponding to 40 to 50 °C and 2.0 M methanol concentration. In addition, a clear trough appears on the right lower fringe, corresponding to 60 °C and 1.0 M methanol as the current density diminishes at higher temperatures and lower methanol concentrations. Those inferences hint at the possibility of tuning the process with the help of controlling the interplaying variables at the desired levels to alleviate the constraints that might be induced by one arbitrary factor level, such as if the electrocatalyst available has undesirable Mo content (i.e., >10 wt.%), which can be compensated by working at a mid-range temperature with a higher concentration of methanol and vice versa.

Regression analysis was conducted to correlate mathematically the optimization parameter expressed in terms of signal-to-noise (S/N) ratios to the factors’ levels. For the model equation to represent the maximal response, the S/N ratio is generated with the “Larger is the better” option, Equation (9).

S/N ratio = −16.3 + 1.144A + 27.28B + 1.92C − 0.1967A2 − 10.62B2 − 0.0330C2 + 0.00559A3 + 1.266B3 + 0.000181C3(9)

where A, B, and C are the control factors representing Mo content, methanol concentration, and reaction temperature, respectively. The model is nonlinear, with an *R*^2^ of 0. 978 and a corrected *R*^2^ of 0.945. In seeking a more simplified form, the cubic terms can be rolled out safely, since the interactions at such a level are deemed insignificant for such processes, Equation (10).

S/N ratio = −16.3 + 1.144A + 27.28B + 1.92C − 0.1967A2 − 10.62B2 − 0.0330C2(10)

To experimentally verify the adequacy of the derived optimal parameters and the mathematical model, a set of experiments has been conducted at previously unexplored conditions. The results are given in Table 5, indicating the presence of some discrepancies, especially at conditions far away from the optimum ones. The differences may be ascribed to the rolling out of the higher-order terms and the failure to derive the interaction terms. However, the model yields predictions with reasonable accuracy at the conditions approaching the optimal ones.

## 4. Conclusions

Electrospun mates composed of poly(vinyl alcohol), nickel acetate, and molybdenum chloride were calcined under a vacuum. The decomposition of the metallic ingredients and graphitization of the used polymer leads to the formation of zero-valent nickel and molybdenum carbide nanoparticles incorporated in amorphous graphite nanofibers. When the Mo content is optimized, the fabricated composite nanofibers demonstrated efficient and stable electrocatalytic activity towards methanol electrooxidation process. The highest rate of methanol electrooxidation with in vivo electrode regeneration can be attained by keeping the molybdenum precursor at 5 wt.% to the nickel acetate in the initial electrospun solution. Calcination temperature proved a critical impact, as the electrooxidation activity toward methanol was enhanced greatly at 850 °C. Employing Taguchi’s robust design helped capture the optimum conditions, yielding the maximum current density: Mo content: 5 wt.%, methanol concentration: 2.65 M, and reaction temperature: 50 °C. Interestingly, the reaction rate proportioned inversely with the medium temperature, as the maximum methanol electrooxidation rate was attained at 45 °C and the optimum was determined as 50 °C from Taguchi’s analysis. Finally, due to its high performance, the proposed Mo_2_C/Ni-incorporated carbon composite in nanofibrous morphology is a highly recommended electrocatalyst for methanol oxidation and should be investigated with other organic substrates.

## Figures and Tables

**Table 1:** Levels of the process parameters.

Variable	Symbol	Unit	Level			
			L1	L2	L3	L4
Mo/Ni content (Mo wt.%)	A	%	5%	10%	15%	25%
Methanol Concentration	B	M	1	2	3	4
Reaction temperature	C	°C	35	45	55	65

**Table 2:** Electrocatalytic activity of reported electrocatalysts toward methanol oxidation.

No	Material	Current Density mA/Cm^2^	Ref.
1	PtFe(1:2)@a-FeOx/NC-C (7. Wt% Pt)	2.34	[65]
2	CNF/PpPD/CuNDs	50	[66]
3	Cu/Rh-SAM-Ni	28	[67]
4	Cu/Rh-SAM	24	[67]
5	Cu bare	22	[67]
6	CPE/CNF/PpPD/NiPs	38.11	[68]
7	NiSe_2_/NC-450	164.68	[69]
8	NiCo_2_S_4_/CNT	160	[70]
9	Ni/PPy/rGO	32.39	[70]
10	Ni/rGO	23.45	[70]
11	PPy/rGO	20	[71]
12	MWCNT/PPy/Pd	45.31	[72]
13	Ni/Cu/Mo@MWCNTs	45	[73]
14	Pt@TiN/rGO	3.09	[73]
15	Pt/rGO	2.01	[73]
16	Pt/Vulcan	1.61	[74]
17	Cu_2_O/PPy-Go	0.3	[75]
18	Pt/PDDA-G	40	[76]
19	PPy(ND)/Au/Cu_2_O	62	[77]
20	Pt/TiO_2_/PTP	0.84	[78]
21	Pt-Ru/C (Commercial)	0.28	[79]
22	Pt/C	2.65	[80]
23	Mo_2_C/Ni-CNFs (5%)	107	Current study

**Table 3:** Design matrix of the factors and their levels utilized for Taguchi analysis.

Exp. No	(Mo) wt.%	(Me), M	Temp., °C	EOP, mA/cm^2^	S/N Ratio
1	5	1	35	80.0	37.7725
2	5	2	45	150.0	43.2119
3	5	3	55	105.0	40.5050
4	5	4	65	108.0	40.7474
5	10	1	45	60.0	35.1687
6	10	2	35	70.0	37.1913
7	10	3	65	70.0	37.1913
8	10	4	55	65.0	36.1204
9	15	1	55	30.0	29.2325
10	15	2	65	39.6	31.6490
11	15	3	35	35.0	30.6183
12	15	4	45	38.0	31.8127
13	25	1	65	25.0	28.6788
14	25	2	55	60.0	35.8967
15	25	3	45	45.0	32.5227
16	25	4	35	35.0	31.2238
F	75.9	11.89	1.9	80.0	
P	0.00	0.006	0.23		
R^2^	97.80				
Rank	1	2	3		
SS	235.36	36.88	5.903		
Contribution, %	82.77	12.97	2.08		

**Table 4:** Analysis of variance SNR (EOP).

Source	DF	Seq SS	Contribution	Adj SS	Adj MS	F-Value	*p*-Value
Mo%	3	235.359	82.77%	235.359	78.453	75.90	0.000
(Me)	3	36.880	12.97%	36.880	12.293	11.89	0.006
T	3	5.903	2.08%	5.903	1.968	1.90	0.230
Error	6	6.202	2.18%	6.202	1.034		
Total	15	284.343	100.00%				

**Table 5:** Comparison of experimental and predicted results.

No.	Experimental				Predicted by Taguchi		% Diff.
	Mo%	Methanol, M	Temp. °C	Peak CD	S/N Ratio	Peak CD	
1	10	1	35	55	33.4	43	21.82
2	10	3	35	70	35.93	58.05	17.07
3	15	1	35	30	27.98	12.5	58.33
4	15	2	35	35	32.19	43.6	−24.57
5	5	3	55	105	41.46	112.6	−7.24
6	5	2.5	50	128	42.8	136	−6.25

## Data Availability

The data presented in this study are available upon request from the corresponding author.
